# Randomized controlled trial of Tesomet for weight loss in hypothalamic obesity

**DOI:** 10.1530/EJE-21-0972

**Published:** 2022-03-16

**Authors:** Kim Huynh, Marianne Klose, Kim Krogsgaard, Jørgen Drejer, Sarah Byberg, Sten Madsbad, Faidon Magkos, Abdellatif Aharaz, Berit Edsberg, Jacob Tfelt-Hansen, Arne Vernon Astrup, Ulla Feldt-Rasmussen

**Affiliations:** 1 Department of Medical Endocrinology and Metabolism PE 2131/2, Copenhagen University Hospital (Rigshospitalet), Copenhagen Ø, Denmark; 2 Saniona A/S, Glostrup, Denmark; 3 Department of Biomedical Sciences, University of Copenhagen, Copenhagen N, Denmark; 4 Department of Endocrinology, Hvidovre University Hospital, University of Copenhagen, Hvidovre, Denmark; 5 Department of Clinical Medicine, Faculty of Health and Clinical Sciences, Copenhagen, Copenhagen N, Denmark; 6 Department of Nutrition, Exercise and Sports (NEXS), University of Copenhagen, Frederiksberg C, Denmark; 7 Department of Endocrinology, Slagelse Hospital, Slagelse, Denmark; 8 Department of Cardiology, The Heart Centre, Copenhagen University Hospital (Rigshospitalet), Copenhagen Ø, Denmark; 9 Department of Forensic Medicine, Faculty of Medical Sciences, University of Copenhagen, Copenhagen, Denmark

## Abstract

**Context:**

Hypothalamic injury often leads to rapid, intractable weight gain causing hypothalamic obesity, which is associated with increased risk of cardiovascular and metabolic morbidity and mortality. There are no approved or effective pharmacological treatments for hypothalamic obesity, and conventional lifestyle management remains ineffective.

**Objective:**

To investigate the safety and efficacy of Tesomet (0.5 mg tesofensine/50 mg metoprolol) in adults with hypothalamic obesity.

**Methods:**

Twenty-one adults with hypothalamic obesity (16 females) were randomized to Tesomet (0.5 mg/50 mg) or placebo for 24 weeks. Patients also received diet/lifestyle counselling. The primary endpoint was safety; secondary endpoints included measures of body weight, appetite scores, quality of life, and metabolic profile.

**Results:**

Eighteen patients completed 24 weeks. Consent withdrawal, eligibility, and serious adverse events (SAE) unrelated to treatment resulted in dropouts. One patient experienced a Tesomet-related SAE of exacerbated pre-existing anxiety leading to treatment discontinuation. Tesomet-related adverse events were otherwise mostly mild and included sleep disturbances (Tesomet 50%, placebo 13%), dry mouth (Tesomet 43%, placebo 0%), and headache (Tesomet 36%, placebo 0%). No significant differences in heart rate or blood pressure were observed between groups. Compared to placebo, Tesomet resulted in additional mean (95% CI) weight change of −6.3% ((−11.3; −1.3); *P*  = 0.017), increased the number of patients achieving ≥5% weight loss (Tesomet 8/13, placebo 1/8; *P*  = 0.046), and tended to augment the reduction in waist circumference by 5.7 cm ((−0.1; 11.5); *P*  = 0.054).

**Conclusion:**

Tesomet was welltolerated, did not affect heart rate or blood pressure, and resulted in significant reductions in body weight compared to placebo in adults with hypothalamic obesity.

## Introduction

Hypothalamic obesity is a complex neuroendocrine disorder characterized by hyperphagia, rapid severe weight gain, reduced basal metabolic rate, and leptin and insulin resistance. Patients face increased risk of developing cardiovascular and metabolic disorders, obstructive sleep apnoea, and non-alcoholic fatty liver disease resulting in increased morbidity and mortality (1, 2, 3, 4, 5). The syndrome is caused by disruption of hypothalamic structures involving satiety and energy homeostasis, often due to hypothalamic injury related to brain tumours and/or their treatment (6, 7). Craniopharyngioma, the most common cause of hypothalamic obesity, has an overall incidence of approximately 1.3–1.7 per million people/year (8, 9). Hypothalamic obesity develops in approximately 50% of craniopharyngioma survivors (10, 11).

There are currently no approved pharmacological treatments for hypothalamic obesity, and conventional weight management (diet and lifestyle modifications) remains mostly ineffective (12, 13). Bariatric surgery may produce sustained weight loss in some patients (14, 15) but is not an option for most because long-term safety and malabsorption issues may present a challenge (16, 17).

Possible impaired sympathoadrenal activity in hypothalamic obesity has led to investigating central stimulants as therapeutic targets (18, 19). Studies of central stimulants (e.g. methylphenidate, dextroamphetamine) or monoamine reuptake inhibitors (sibutramine) reported encouraging results for managing weight gain in these patients, but for the latter, concern over potential long-term cardiovascular side effects prevented widespread use (20, 21, 22, 23, 24, 25). Tesofensine (NS2330) is a centrally acting monoamine reuptake inhibitor with a more balanced monoamine effect and substantially different pharmacokinetic profile than sibutramine. Tesofensine results in appetite suppression and weight loss by inhibiting the presynaptic reuptake of dopamine, serotonin, and noradrenaline (26, 27). The pharmacodynamic profile of tesofensine has not been fully elucidated, but rodent studies suggested that indirect α_1_-adrenergic and D_1_-dopaminergic stimulation contribute to its appetite-suppressing and weight-reducing effects (28). Tesofensine was initially developed for Parkinson’s and Alzheimer’s diseases. Although ineffective at treating these neurodegenerative diseases, a noted side effect was weight loss (29, 30). This was later confirmed in patients with general obesity and was accompanied by not only favourable changes in blood lipids but also increased heart rate at therapeutic doses and mild and sporadic blood pressure increases at higher doses (31). These cardiovascular side effects of tesofensine were prevented in obese rats when co-administered with the selective beta-1-blocker, metoprolol, while still inhibiting food intake and promoting weight loss (32). Co-administering tesofensine and metoprolol in a 1:100 dose ratio also counteracted tesofensine-induced increased heart rate at doses ≤0.75 mg in a phase 1 clinical trial (NCT03488719) (Saniona A/S, unpublished results) in otherwise healthy individuals with overweight or obesity.

The present study investigated the safety and efficacy of Tesomet (0.5 mg tesofensine/50 mg metoprolol) in adults with hypothalamic obesity. We hypothesized that treatment with Tesomet would suppress appetite and produce weight loss without cardiovascular side effects.

## Subjects and methods

### Study design

In a phase 2, double-blind, randomized, placebo-controlled, interventional trial, 21 adult hypopituitary patients with hypothalamic obesity (16 females) were recruited from a tertiary referral centre (Department of Endocrinology and Metabolism, Rigshospitalet, Copenhagen, Denmark) and randomized to Tesomet (0.5 mg tesofensine/50 mg metoprolol) or placebo daily for 24 weeks. Both groups were instructed to follow a hypocaloric diet (energy deficit of 300 kcal daily) and given monthly lifestyle counselling from a trained dietician.

The primary objective was safety evaluated by number and type of treatment-emergent adverse events, vital signs, biochemical data, 24-h ambulatory blood pressure measurement, Holter monitoring, and ECG. Adverse events were classified as mild, moderate, or severe according to the Medical Dictionary for Regulatory Activities (MedDRA, version 22.0). Efficacy measures included change from baseline to week 24 in anthropometry, body composition, and subjective appetite scores, self-reported health-related quality of life (QoL), and lipid and glucose profile. An overview of methods and measurements is listed in Table 1.

**Table 1:** Measurements and methods for a randomized clinical trial of Tesomet for hypopituitary patients with hypothalamic obesity.

Measurement/assessment (unit)	Method
Adverse events	
Mild/moderate/severe	MedDRA, version 22.0
Anthropometry^	
Height (cm)	To the closest 0.1 cm using a wall-mounted stadiometer*
Weight (kg)	To the closest 0.1 kg on a pre-calibrated scale**
Waist (cm)	To the nearest 1.0 cm midway between lower costal edge and upper iliac crest.
Body composition^^	A trained operator performed all scans, Cv_max_ <1% for all DXA outcomes.
Fat mass (kg)	
Lean mass (kg)	
Bone mineral content (g)	
Bone mineral density (g/cm^2^)	
Biochemical assessment^^^	
Total cholesterol (mmol/L)	Cobas CHOL 2 agent, CV_max_ 5% ***
HDL-cholesterol (mmol/L)	Cobas HDLC4 agent, CV_max_ 4% ***
LDL-cholesterol (mmol/L)	Cobas LDLC3 agent, CV_max_ 4% ***
Triglyceride (mmol/L)	Cobas TRIGL agent, CV_max_ 4% ***
Plasma glucose (mmol/L)	Cobas GLUC3 agent, CV_max_ 4% ***
HbA1c (mmol/mol)	Ion-exchange HPLC, CV_max_ 4%****
Questionnaires	
Short form 36 v.2^#^	Physical component score and a mental component score using a standardized algorithm (37). Higher scores indicate higher self-perceived QoL.
PHQ-9^##^	Total depression score. The questionnaire scores nine depression DSM-IV criteria from 0 (not at all) to 3 (nearly every day).
Appetite (mm)	Visual analogue scale (100 mm). Appetite scores were summarized into a composite satiety score (CSS). Higher CSS represents a more full, satiated state with less hunger and lower prospective food consumption.
Food cravings (mm)	Visual analogue scale (100 mm). High values represent a low self-perceived desire for the food element in question.

^Anthropometry measurements were performed by a single trained staff member; ^^Full-body DXA scanner (ME+200588, GE Healthcare A/S), analysed using proprietary GE CoreScan software (GE Healthcare); ^^^Fasting venous blood samples were drawn for laboratory analyses at each visit; #Short Form 36 Health Survey Questionnaire version 2 (SF-36v2, Health Survey Standard, Denmark, Danish) (35, 36). ##Patient Health Questionnaire 9 (Danish version) (51, 52); *Without shoes in upright standing position with the back to the wall down (Seca stadiometer, Kirudan, Broendby, Denmark); **Weight was measured without shoes, in light clothing; ***Cobas 8000 c702 module photometric apparatus (Roche Diagnostics GmbH); ****Tosoh G8 analyzer (Sysmex Europe GmbH, Hamburg, Germany).

### Patients

Eligible patients were males and females aged 18–75 with BMI ≥27 kg/m^2^ who had experienced abrupt unexplained weight gain related to hypothalamic injury as judged by the treating physician (either before neurosurgical removal of a large mass proximal to the hypothalamus, post-neurosurgery, or a slower progression of weight gain in case of cranial irradiation). Additional inclusion and exclusion criteria are listed in Table 2. The study protocol was approved by the Capital Region Scientific Ethics Committee (H-18058856) (Hilleroed, Denmark), and the Danish Medicine Agency. All patients provided informed consent. The trial was conducted in compliance with the International Conference on Harmonisation/Good Clinical Practice (ICH/GCP) guidelines and registered at www.clinicaltrials.gov (NCT03845075).

**Table 2:** Study inclusion and exclusion criteria for a randomized clinical trial of Tesomet for hypopituitary patients with hypothalamic obesity.

Inclusion criteria
Age ≥18 and ≤75 years BMI ≥27 kg/m^2^ Hypothalamic injury-related weight gain Normal blood pressure or well-managed hypertension for >2 months Well-managed and stable hypopituitarism for >2 months
Exclusion criteria
Hypersensitivity to Tesomet or its components Abnormal metabolic conditions: type 1 diabetes, Cushing’s syndrome, acromegaly, hypophysitis, Prader–Willi syndrome Untreated hypo- or hyperthyroidism suicidal indentation or behaviour Clinically significant liver or kidney impairment ≥5% weight loss within the last 3 months Pregnant or lactating women Failure to comply with adequate contraceptive methods NYHA^a^ class ≥2 Myocardial arrhythmias Myocardial infarction or stroke within 5 years Blood pressure ≥160/90 mmHg Heart rate ≥90 or <50 bpm Patients with type 2 diabetes were excluded if HbA1c ≥86 mmol/mol (10%) or fasting plasma glucose ≥11 mmol/L (198 mg/dL). Patients using glucagon-like peptide-1 (GLP-1) analogues for the treatment of type 2 diabetes had to be on a stable dose for >3 months. Psychiatric diagnosis Suicidal indentation or behaviour Clinically significant patient health questionnaire-9 (PHQ-9) score Treatment with beta-blockers or monoaminoxidase-inhibitors Eating disorders Enrolment in another clinical study

^a^New York Heart Association (NYHA) Functional Classification.

HbA1c, haemoglobin A1c.

### Procedure

**Figure 1:**
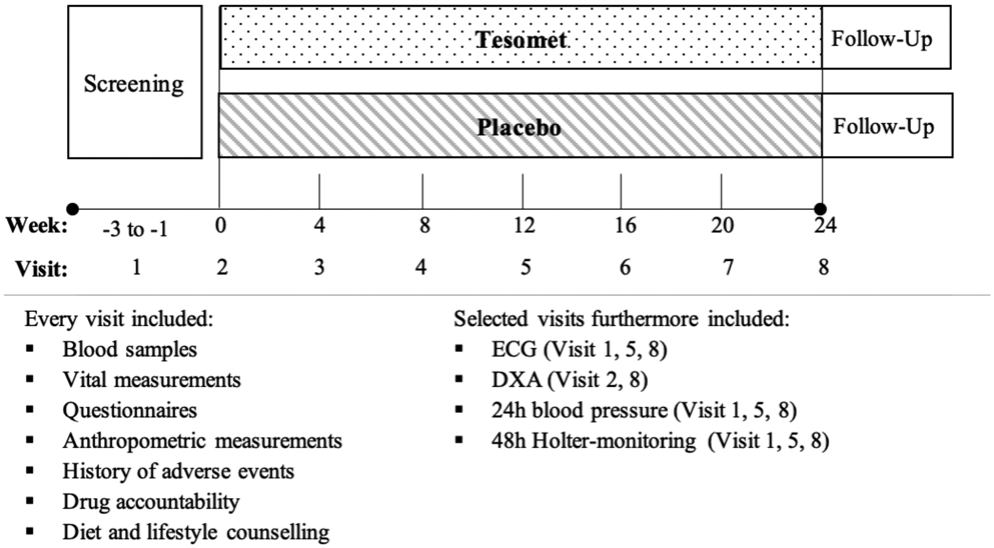
Trial timeline of a randomized controlled trial of Tesomet for weight loss in with hypothalamic obesity. Outline of the trial procedures. Telephone consultations were performed between each on-site visit. Follow-up to address adverse events after end of treatment was performed 45 ± 3 days after the last dose of investigational product. DXA, dual-energy X-ray absorptiometry.

Following screening, participants were scheduled for a baseline visit, and six on-site follow-up visits 28 *±*3 days apart with telephone visits in-between (Fig. 1). At baseline, patients were allocated the first available randomization number by an external statistician from a computer-generated list to ensure 2:1 (Tesomet:placebo) randomization of patients without diabetes and a 1:1 randomization of patients with type 2 diabetes. Patients fasted overnight prior to on-site visits conducted in the morning hours. Fasting was defined as at least 8 h without food and fluid intake, except water and prescribed medication other than trial product and glucose-lowering agents. Visits included clinical assessment, adverse events, blood sampling and questionnaires regarding appetite, QoL (SF-36), and depression (PHQ-9). A dual-energy X-ray absorptiometry (DXA) scan was performed at baseline and week 24. ECG, 24-h ambulatory blood pressure, and 48-h Holter monitoring were performed at screening, week 12, and week 24. A set of predefined criteria were used to evaluate the safety of patient continuation in the trial (see study protocol section 3.10 (clinicaltrials.gov)). Tesomet and placebo packaging and appearance were identical. Patients attended a counselling session with a trained dietician after each on-site visit. The dietician instructed patients to follow a low-calorie, moderately low-fat diet (30% total energy from fat, 20% protein, and 50% carbohydrates), designed to provide a daily 300 kcal energy deficit based on estimated total energy requirements for each individual patient. Patients were instructed to follow official Danish dietary recommendations and change unhealthy lifestyle habits and were encouraged to increase physical activity to ≥30 min/day on most days of the week.

### Appetite and food cravings

Ratings of hunger, satiety, fullness, and prospective food consumption, as well as cravings for sweet, salty, savoury, and fatty foods, were assessed using visual analogue scales (VAS, 100 mm in length) (33, 34). Appetite scores were summarized into a composite satiety score (CSS) using the formula:


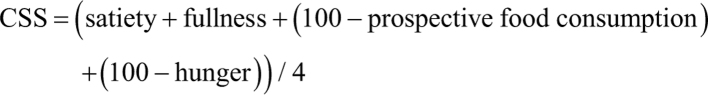


Higher composite satiety scores represent a more full, satiated state with less hunger and lower prospective food consumption. Scales for cravings of different food elements were constructed where high values represent a low self-perceived desire for the food element in question. Patients were instructed to complete these scales at each visit conducted in the morning after consuming a standardized breakfast.

### Health-related quality of life

The SF-36 is a well-validated 36-item questionnaire, comprehensively evaluating both physical and mental health (35, 36) using a standardized algorithm (37). Higher scores indicate higher self-perceived QoL in the corresponding domains.

### Statistical analysis

Statistical computations were made in SAS (version 9.3 or later) (SAS Institute Inc., Cary, NC, USA) and SPSS Statistics, version 25.0 (IBM Corp). The safety population included all randomized patients receiving at least one dose of investigational drug. The modified intention-to-treat (mITT) population included all randomized patients with non-missing baseline data and at least one post-baseline measurement. Analyses were based on the mITT population having the last observation carried forward (LOCF) for missing data unless otherwise stated. Endpoint data were compared between treatment groups using a baseline-adjusted analysis of covariance (ANCOVA) model with change from baseline as the dependent factor, treatment group as the independent factor, and baseline-value as covariate. Results are presented as placebo-subtracted mean difference ((95% CI), *P* -value) unless otherwise stated. Linear regression was performed to explore correlations between endpoint measures. Regression analysis only included observed data. Results are presented as r-squared (*r* ^2^) and *P* -value. Alpha was set at *P*  < 0.05.

## Results

### Participants

**Figure 2:**
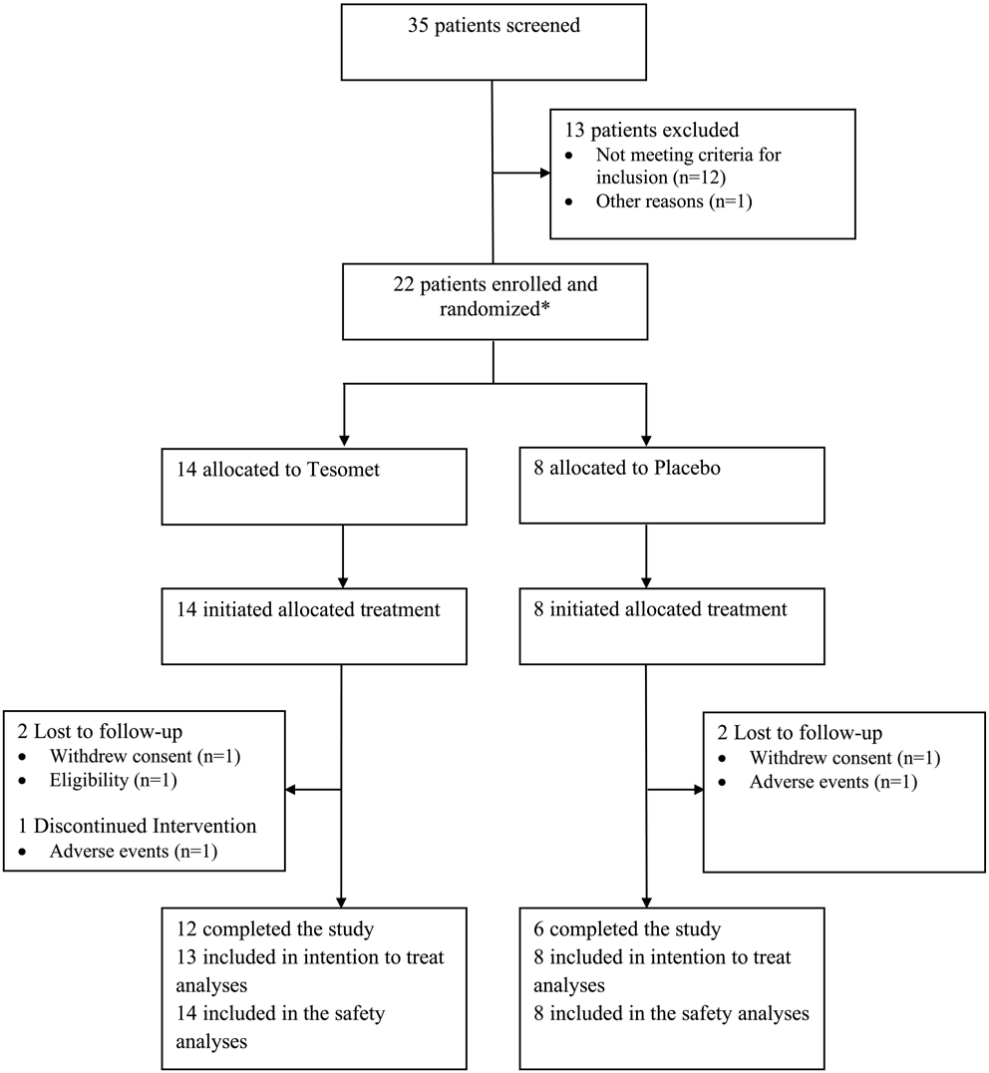
Consort diagram of a randomized controlled trial of Tesomet for weight loss in with hypothalamic obesity. Consort flowchart illustrating the process of recruitment, allocation, follow-up, and analyses. *Including one patient who was randomized twice in error (see ‘Participants’ section in ‘Results’ section).

In total, 35 patients were screened, of whom 21 unique patients (16 females) fulfilled eligibility criteria and were randomized (Fig. 2). One female patient screen failed but was randomized in error and received a single dose of Tesomet but completed no post-dose assessments. This patient was 78 days later re-screened and randomized to placebo. Patients were balanced between treatment groups at baseline (Table 3). Fifteen patients had panhypopituitarism and 11 received desmopressin. Hypopituitarism was pharmacologically managed in all but three female patients randomized to placebo who opted not to have their hypogonadism or growth hormone deficiency substituted.

**Table 3:** Patient demographics and baseline characteristics in a randomized clinical trial of Tesomet for hypopituitary patients with hypothalamic obesity. Data presented as mean ± s.d. or *n* (% of total) for each treatment group. No statistically significant differences using Student’s *t*-test for continuous variables or Fisher’s exact test for categorical variables were found.

	Tesomet (*n* =14)	Placebo (*n* =8)
Age (years)	45.4 ± 13.3	44.4 ± 18.3
Sex, *n* (%)		
Female	11 (78.6%)	6 (75%)
Male	3 (21.4%)	2 (25%)
White or Caucasian n (%)	14 (100%)	8 (100%)
Not Hispanic or Latino n (%)	14 (100%)	8 (100%)
Time from assumed hypothalamic injury (years)	12.0 ± 8.3	20.3 ± 15.7
Age at initial treatment (years)	33.9 ± 16.9	24.6 ± 16.4
Height (cm)^a^	175.1 ± 7.6	171.3 ± 10.8
Weight (kg)^a^	114.3 ± 18.1	112.2 ± 27.0
Waist circumference (cm)^a^	117.5 ± 12.2	116.1 ± 17.9
BMI (kg/m^2^)^a^	37.3 ± 5.6	37.8 ± 5.8
DXA		
Fat mass (kg)^a^	51.7 ± 15.0	48.6 ± 13.3
Lean mass (kg)^a^	58.7 ± 11.5	59.7 ± 19.2
Tumour type, *n* (%)^b^		
Craniopharyngioma	6 (42.9%)	4 (50%)
Adamantinomatous	4 (28,6%)	2 (25%)
Papillary	2 (14,3%)	0
Unknown^c^	0	2 (25%)
Pituitary Macroadenoma^d^	4 (28.6%)	1 (12.5%)
Astrocytoma	2 (14.3%)	1 (12.5%)
Meningioma	1 (7.1%)	1 (12.5%)
Glioma	0	1 (12.5%)
Germinoma	1 (7.1%)	0
Tumour treatment, *n* (%)		
Neurosurgery	13 (92.9%)	6 (75%)
Irradiation therapy	9 (64.3%)	3 (37.5%)
Chemotherapy	2 (14.3%)	1 (12.5%)
Endocrinopathy, *n* (%)		
Central hypothyroidism	14 (100%)	8 (100%)
Central adrenal insufficiency	13 (92.9%)	6 (75%)
Hypogonadotropic hypogonadism	11 (78.6%)	6 (75%)
Diabetes insipidus	7 (50.0%)	4 (50%)
Growth hormone deficiency	11 (78.6%)	5 (62.5%)
Diabetes mellitus type 2	2 (14.3%)	1 (12.5%)

^a^Data unavailable for one patient randomized to Tesomet. ^b^Diagnosis confirmed by histological or radiological presentation. ^c^Histological type could not be determined from medical history. ^d^Giant macroadenomas; three of four patients (Tesomet) had undergone several pituitary surgeries + subsequent irradiation, whereas one patient (Placebo) had only undergone surgery. This patient developed an SAE (hyponatraemia) and withdrew from the trial.

Eighteen patients completed 24 weeks of treatment. Tesomet discontinuation was secondary to anxiety (*n* = 1), consent withdrawal (*n* = 1), and eligibility (*n* = 1), while placebo discontinuation was due to symptomatic hyponatraemia (*n* = 1) and finding study procedures too demanding (*n* = 1). The patient who developed anxiety continued the trial without investigational treatment, whereas the four remaining patients dropped out. The patient randomized twice, once in error to Tesomet and later to placebo, was included in both the Tesomet and placebo data sets for the safety analyses (*n* = 22) but was only included in the placebo arm for the mITT analysis (*n* = 21).

### Safety

Three patients experienced serious adverse events (SAEs); two randomized to Tesomet and one to placebo. In the Tesomet group, one patient developed anxiety related to Tesomet and the other had recurrence of craniopharyngioma with subsequent post-procedural complications to surgery unrelated to Tesomet. In the placebo group, one patient developed symptomatic hyponatraemia. All SAEs resolved. In total, 64 adverse events (AE) were recorded in 12 (86%) patients randomized to Tesomet. The most common AEs were sleep disturbances, dry mouth, headache, and dizziness (Table 4). The prevalence and severity of adverse events was similar between groups.

**Table 4:** Adverse events in the safety population of a randomised clinical trial of Tesomet for hypopituitary patients with hypothalamic obesity by System Organ Class and Preferred Medical Term. Data presented as no. patients with event (% of patients) no. events for each treatment group in the safety population. The number of patients in each subgroup was too small to perform inferential tests.

	Tesomet (*n* = 14)		Placebo (*n* =8)	
	Patients, *n* (%)	Events, *n*	Patients, *n* (%)	Events, *n*
All adverse events	12 (86%)	64	7 (88%)	34
Serious adverse events	2 (14%)	3	1 (13%)	1
Treatment discontinuation adverse events	1 (7%)	2	1 (13%)	1
Severity of adverse events				
Mild	11 (79%)	36	7 (88%)	14
Moderate	10 (71%)	23	5 (63%)	17
Severe	2 (14%)	5	2 (25%)	3
Gastrointestinal disorders	9 (64%)	17	5 (63%)	12
Abdominal pain, upper	3 (21%)	3	3 (38%)	5
Dry mouth	6 (43%)	6	0	
Nausea	2 (14%)	3	2 (25%)	2
Vomiting	1 (7%)	1	2 (25%)	2
Constipation	2 (14%)	2	0	
Diarrhoea	0		2 (25%)	2
Faeces hard	2 (14%)	2	0	
Gastroesophageal reflux disorders	0		1 (13%)	1
Nervous system disorders	8 (57%)	16	3 (38%)	6
Dizziness	6 (43%)	9	3 (38%)	6
Headache	5 (36%)	6	0	
Presyncope	1 (7%)	1	0	
Psychiatric disorders	7 (50%)	9	1 (13%)	1
Sleep disorders	7 (50%)	7	1 (13%)	1
Anxiety*	1 (7%)	1	0	
Paranoia*	1 (7%)	1	0	
Musculoskeletal and connective tissue disorders	3 (21%)	3	2 (25%)	2
Muscle spasms	1 (7%)	1	0	
Musculoskeletal pain	1 (7%)	1	0	
Myalgia	0		1 (13%)	1
Neck pain	1 (7%)	1	0	
Pain in extremity	0		1 (13%)	1
Skin and s.c. tissue disorders	3 (21%)	3	1 (13%)	1
Hyperhidrosis	3 (21%)	3	0	
Night sweats	0		1 (13%)	1
General disorders and administration site conditions	2 (14%)	2	2 (25%)	2
Fatigue	1 (7%)	1	1 (13%)	1
Pyrexia	0		1 (13%)	1
Energy increased	1 (7%)	1	0	
Cardiac disorders	0		1 (13%)	1
Palpitations	0		1 (13%)	1
Metabolism and nutritional disorders	0		1 (13%)	1
Hyponatremia	0		1 (13%)	1
Investigational	1 (7%)	1	0	
Blood pressure increased	1 (7%)	1	0	
Neoplasms	1 (7%)	1	0	
Craniopharyngioma	1 (7%)	1	0	
Injury, poisoning and procedural complications	1 (7%)	1	1 (13%)	1
Fall	0		1 (13%)	1
Post-procedural complications	1 (7%)	1	0	
Hepatobiliary disorders	0		1 (13%)	1
Cholecystitis	0		1 (13%)	1
Respiratory, thoracic and mediastinal disorders	2 (14%)	3	1 (13%)	1
Cough	0		1 (13%)	1
Rhinorrhoea	1 (7%)	2	0	
Viral upper respiratory tract infection	1 (7%)	1	0	
Infections and infestations	6 (43%)	8	3 (38%)	5
Gastroenteritis	1 (7%)	1	1 (13%)	1
Viral tonsillitis	0		2 (25%)	2
Viral upper respiratory tract infection	2 (14%)	2	0	
Upper respiratory tract infection	1 (7%)	1	0	
Eye infection	1 (7%)	1	0	
Gastroenteritis viral	0		1 (13%)	1
Gingivitis	1 (7%)	1	0	
Tonsillitis	1 (7%)	1	0	
Influenza	0		1 (13%)	1
Sinusitis	1 (7%)	1	0	

*One patient developed both anxiety and paranoia.

One Tesomet patient had increased gamma-glutamyl transferase (+181 U/L) and alkaline phosphatase (+47 U/L) at week 24; the patient was diagnosed with sinusitis and had started antibiotic treatment 7 days earlier. Both values spontaneously normalized at the next scheduled visit. Laboratory safety data did not otherwise differ between groups at week 24 (Table 5). Change in blood pressure (including 24-h ambulatory blood pressure; data not shown) and heart rate did not differ significantly between treatment groups at any time point (Table 6). No cardiac arrhythmias were observed.

**Table 5:** Twenty-four-week observed change in laboratory safety data in the safety population of a randomized clinical trial of Tesomet for hypopituitary patients with hypothalamic obesity. Data are presented as observed mean (95% CI) change from baseline to week 24 in laboratory safety data for each treatment group in the safety population. Only 12 and 6 patients, respectively, completed the study and could be evaluated.

	Tesomet (*n* = 12)	Placebo (*n* = 6)
ALT (U/L)	−1.5 (−6.7; 3.7)	−3.7 (−20.8; 13.4)
AST (U/L)	1.6 (−1.3; 4.4)	0 (−5.7; 5.7)
ALP (U/L)	4.1 (−7.3; 15.5)	−5.2 (−11.9; 1.5)
GGT (U/L)	16.0 (−19.6; 51.6)	−2.3 (−7.5; 2.9)
Haematocrit (%)	0 (−0.1; 0)	0 (−0.1; 0)
Lymphocytes (E9/L)	0.2 (−0.1; 0.4)	0.4 (−0.3; 1.1)
Leukocytes (E9/L)	0.2 (−0.6; 1.0)	−0.1 (−1.2; 1.8)
Creatinine (µmol/L)	0.4 (−3.4; 4.3)	4.8 (−1.7; 11.4)
Sodium (mmol/L)	0.3 (−1.5; 2.2)	0.3 (−3.0; 3.7)
Potassium (mmol/L)	−0.1 (−0.3; 0.1)	−0.2 (−0.5; 0.1)

ALT, alanine aminotransferase; AST, aspartate aminotransferase; ALP, alkaline phosphatase; GGT, gamma glutamyl transferase.

**Table 6:** Twenty-four-week change in outcomes in the modified intention-to-treat population of a randomized clinical trial of Tesomet for hypopituitary patients with hypothalamic obesity. Data presented as least squares mean (95% CI) change from baseline to week 24 in the mITT population. Last observation carried forward imputation was used for missing data. Pair-wise comparisons were performed using a baseline adjusted ANCOVA model with treatment as factor and change from baseline as dependent variable.

	Tesomet (*n* = 13)	Placebo (*n* = 8)	Est. difference: Tesomet vs placebo	*P*
Body weight (kg)	−7.4 (−10.8; −3.9)	−0.4 (−4.9; 4.0)	−6.9 (−12.6; −1.3)	0.02
Body weight (%)	−6.6 (−9.7; −3.5)	−0.3 (−4.3; 3.6)	−6.3 (−11.3; −1.3)	0.02
Waist circumference (cm)	−6.5 (−10.1; −3.0)	−0.9 (−5.4; 3.7)	−5.7 (−11.5; 0.1)	0.054
Waist circumference (%)	−5.7 (−8.8; −2.5)	−0.6 (−4.6; 3.4)	−5.0 (−10.1; 0.0)	0.052
BMI (kg/m^2^)	−2.4 (−3.6; −1.2)	−0.1 (−1.7; 1.4)	−2.3 (−4.2; -0.3)	0.03
DXA^a^				
Fat mass (kg)	−5.3 (−8.2; -2.3)	−1.1 (−5.3; 3.1)	−4.2 (−9.3; 1.0)	0.10
Lean mass (kg)	−2.8 (−3.8; −1.7)	0.4 (−1.2; 1.9)	−3.1 (−5.0; −1.2)	0.003
Bone mineral content (g)	−5.0 (−38.8; 28.8)	−14.7 (−63.6; 34.2)	9.7 (−51.4; 70.9)	0.74
Bone mineral density (g/cm^2^)	0.0 (−0.0; 0.0)	0.0 (−0.0; 0.0)	0.0 (−0.02; 0.02)	0.99
Vital signs variables				
Systolic blood pressure (mmHg)	2.2 (−4.6; 9.0)	−3.7 (−12.5; 5.2)	5.9 (−5.8; 17.5)	0.30
Diastolic blood pressure (mmHg)	0.1 (−5.4; 5.6)	−1.1 (−8.1; 6.0)	1.2 (−7.9; 10.2)	0.79
Heart rate (bpm)	−2.9 (−8.7; 2.8)	−5.6 (−13.0; 1.7)	2.7 (−6.7; 12.1)	0.56
Corrected QT-interval (ms)^a^	−4.2 (−12.9; 4.4)	−3.2 (−15.4; 9.0)	−1.1 (−16; 13.9)	0.88
Cholesterol^a^				
Total (mmol/L)	−0.2 (−0.5; 0.1)	−0.1 (−0.6; 0.3)	−0.05 (−0.59; 0.50)	0.86
LDL (mmol/L)	−0.2 (−0.5; 0.0)	−0.2 (−0.6; 0.2)	−0.06 (−0.53; 0.42)	0.81
HDL (mmol/L)	0.0 (−0.1; 0.2)	0.0 (−0.2; 0.2)	0.03 (−0.25; 0.30)	0.83
Triglycerides (mmol/L)	−0.1 (−0.6; 0.4)	−0.3 (−1.0; 0.4)	0.23 (−0.62; 1.07)	0.58
FPG (mg/dL)^b^	−6.7 (−10.6; −2.8)	−5.8 (−11.2; −0.5)	−0.85 (−7.58; 5.88)	0.79
FPG (mmol/L)^b^	−0.4 (−0.6; −0.2)	−0.3 (−0.6; 0)	−0.05 (−0.42; 0.33)	0.79
HbA1c (%)^a^	−0.5 (−0.6; −0.3)	−0.2 (−0.4; 0.1)	−0.28 (−0.58; 0.03)	0.07
HbA1c (mmol/mol)^a^	−5.1 (−7.0; −3.2)	−2.0 (−4.7; 0.7)	−3.02 (−6.34; 0.30)	0.07

^a^Data available for 12 patients in Tesomet; 6 in Placebo. ^b^Data unavailable for 1 patient randomized to placebo.

ANCOVA, analysis of covariance; FPG, fasting plasma glucose; HbA1c, haemoglobin A1c; HDL, high-density lipoprotein; LDL, low-density lipoprotein; mITT, modified intention-to-treat.

### Efficacy

**Figure 3:**
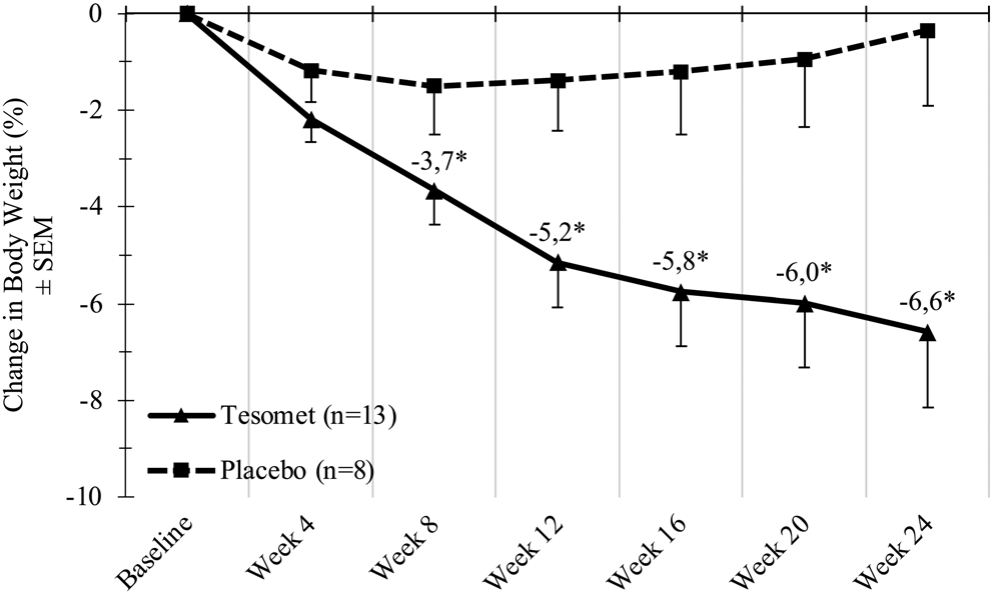
Change in body weight over time in a randomized controlled trial of Tesomet for weight loss in with hypothalamic obesity. Data are mean change from baseline in body weight (%) for each treatment group at each scheduled visit (weeks from baseline). Last observation carried forward imputation was used for missing data. Error bars represent s.e.m. *P  < 0.05 in a baseline adjusted ANCOVA model with treatment as factor.

**Figure 4:**
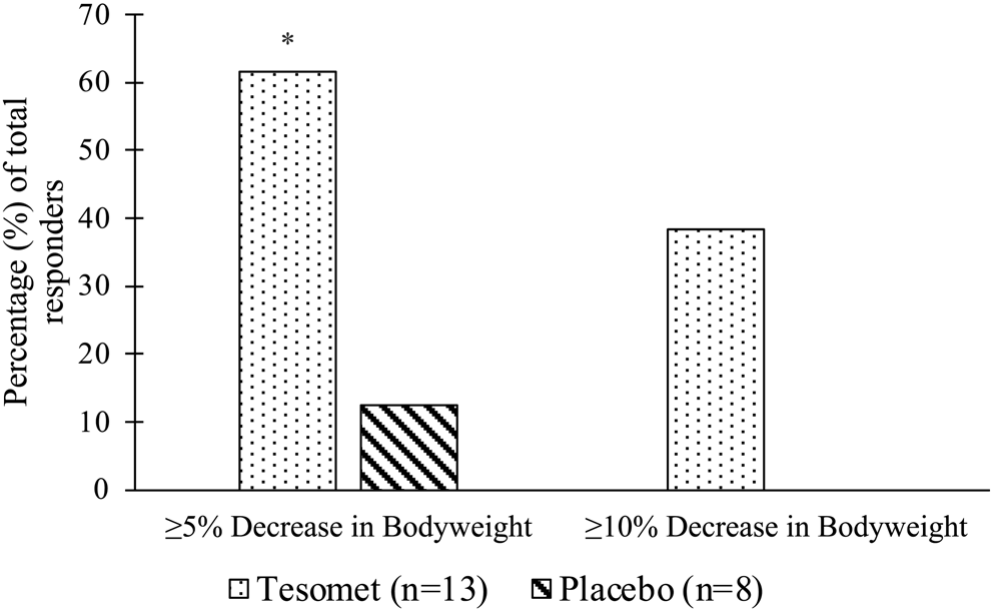
Bar diagram of relative (%) change in a randomized controlled trial of Tesomet for weight loss in with hypothalamic obesity. Relative percentage (%) of patients randomized to Tesomet (*n* = 13) or placebo (*n* = 8) achieving 5% or 10% weight loss from baseline at week 24. *P  < 0.05 in a logistic regression model with treatment as factor.

Weight loss was observed in both groups but was significantly greater in Tesomet-treated compared to placebo-treated patients, starting from week 8 (Fig. 3). At week 24, least squares (LS) mean (95% CI) weight change in patients randomized to Tesomet was −6.6% (−9.7; −3.5) compared to placebo −0.3% (−4.3; 3.6); thus, Tesomet resulted in an additional mean weight change of −6.3% ((−11.3; −1.3), *P*  = 0.02 (Table 6). Tesomet-induced weight loss was more rapid during the first 12 weeks of treatment and did not plateau at any time point (Fig. 3). The number of patients achieving ≥5% reduction in body weight from baseline at week 24 was 8/13 (61.5%) in Tesomet-treated patients and 1/8 (12.5%) in placebo-treated patients, corresponding to an odds-ratio of 11.2 ((1.0; 120.4), *P*  = 0.046). Furthermore, 5/13 (38%) Tesomet-treated patients achieved ≥10% reduction in body weight at week 24, compared to no placebo-treated patients (Fig. 4).

**Figure 5:**
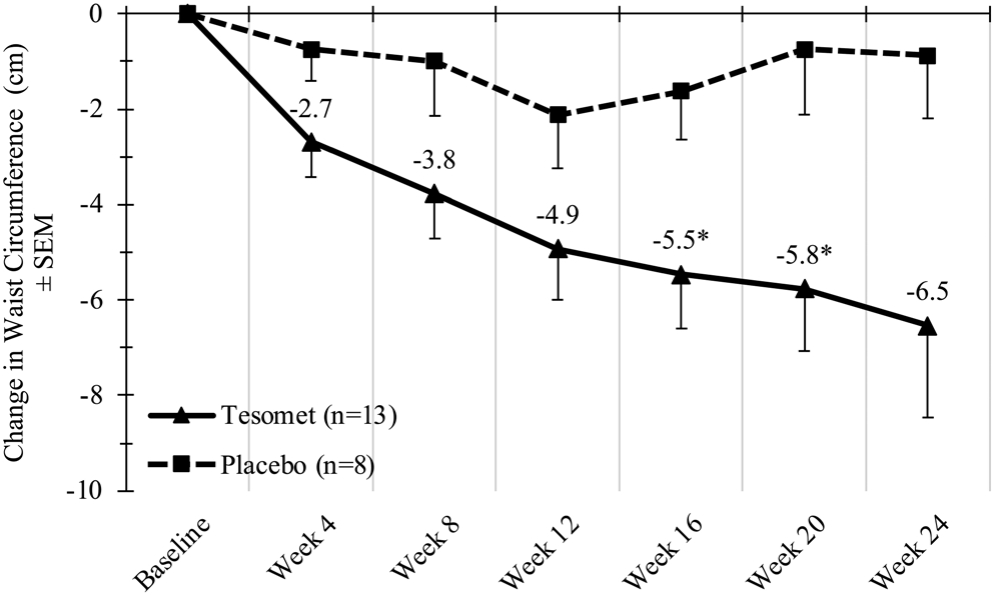
Change in waist circumference over time in a randomized controlled trial of Tesomet for weight loss in with hypothalamic obesity. Data are mean change from baseline in waist circumference (cm) for each treatment group at each scheduled visit (weeks from baseline). Last observation carried forward imputation was used for missing data. Error bars represent s.e.m. *P  < 0.05 in a baseline adjusted ANCOVA model with treatment as factor.

Tesomet-induced weight loss at week 24 was accompanied by a placebo-subtracted mean difference in fat mass of −4.2 kg ((−9.3; 1.0), *P*  = 0.10) and lean mass of −3.1 kg ((−5.0; −1.2), *P*  = 0.003). Consistently, Tesomet resulted in additional mean change in waist circumference of −5.7 cm ((−11.5; 0.1], *P*  = 0.054) over placebo (Fig. 5).

Improvements in metabolic variables were numerically small and not statistically significant (Table 6) and were driven by two Tesomet-treated patients with type 2 diabetes. These two patients exhibited reductions in HbA1c from a baseline of 69 mmol/mol to week 24 of 39 mmol/mol and 85 mmol/mol, to 39 mmol/mol, respectively, representing a mean reduction of 48.8%. Tesomet treatment did not result in changed HbA1c levels in patients without diabetes mellitus (mean ± s.d.: 0.4 mmol/mol ± 1.4) (0.0% ± 0.1).

In univariate regression analyses, Tesomet-induced weight loss correlated with reduced fat mass (*r*^2^= 0.91, *P* ≤ 0.0001), waist circumference (*r*^2^= 0.62, *P*  = 0.002), lean mass (*r*^2^= 0.40, *P*  = 0.03), triglyceride concentrations (*r*^2^= 0.34, *P*  = 0.048), systolic blood pressure (*r*^2^= 0.34, *P*  = 0.048), and heart rate (*r*^2^= 0.34, *P*  = 0.049).

### Satiety and QoL

**Figure 6:**
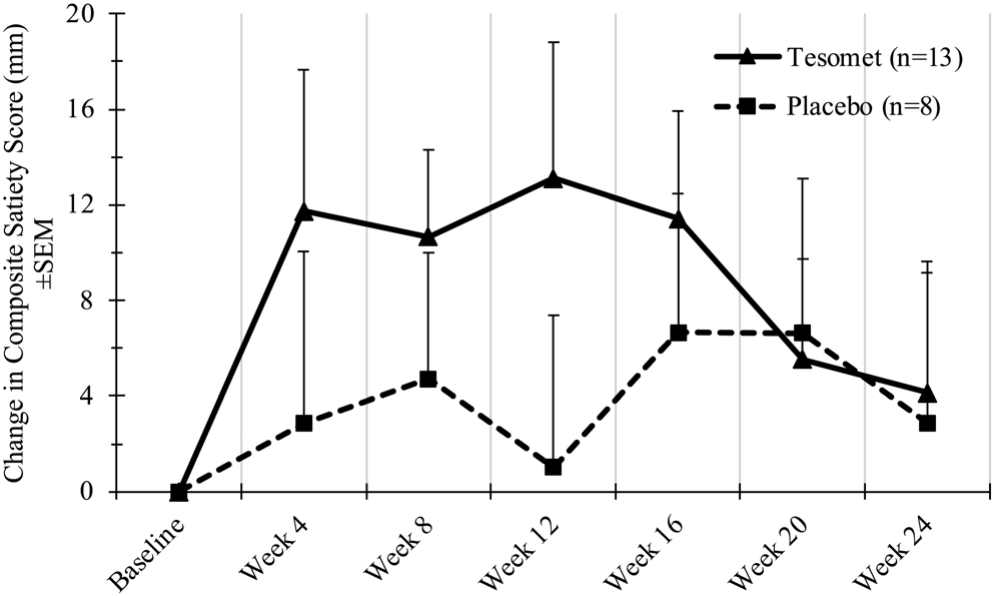
Change in composite satiety score over time in a randomized controlled trial of Tesomet for weight loss in with hypothalamic obesity. Data are mean change from baseline in composite satiety score (mm) for each treatment group at each scheduled visit (weeks from baseline). Last observation carried forward imputation was used for missing data. Error bars represent s.e.m.

Change in the composite satiety score was numerically larger in Tesomet-treated patients during the first 16 weeks but declined to levels near placebo from week 20 (Fig. 6). Changes in satiety and food cravings were not significantly different between groups (Table 7).

**Table 7:** 12- and 24-week change in questionnaire data: appetite, food cravings, and health-related quality of life in the modified intention-to-treat population of a randomised clinical trial of Tesomet for hypopituitary patients with hypothalamic obesity. Data presented as least squares mean (95% CI) change from baseline to week 12 and 24 in the mITT population. Last observation carried forward imputation was used for missing data. Inferential tests were performed using a baseline adjusted ANCOVA model with treatment as factor and change from baseline as dependent variable.

	∆Week 0–12			∆Week 0–24		
	Tesomet (*n* = 13)	Placebo (*n* = 8)	*P*	Tesomet (*n* = 13)	Placebo (*n* = 8)	*P*
Composite satiety score (mm)	12.7 (1.7; 23.8)	1.7 (−12.4; 15.7)	0.21	3.6 (−6.1; 13.4)	3.8 (8.7; 16.2)	0.99
How much do you think you can you eat?	−15.7 (−29.2; −2.2)	−10.2 (−27.5; 7.0)	0.60	−8.3 (−18.1; 1.6)	−12.5 (−25.1; 0.1)	0.59
How full do you feel?	10.1 (−2.1; 22.3)	−1.7 (−17.2; 13.8)	0.22	0.5 (−11.7; 12.6)	8.4 (−7.1; 23.9)	0.41
How hungry do you feel?	−11.7 (−26.4; 3.0)	−2.1 (−20.8; 16.6)	0.41	−5.8 (−18.9; 7.3)	10.1 (−6.6; 26.8)	0.13
How satisfied do you feel?	13.5 (1.5; 25.6)	−4.4 (−19.8; 11.1)	0.07	0.6 (−11.8; 12.9)	3.2 (−12.5; 19.0)	0.78
How comfortable do you feel?	1.5 (−10.7; 13.7)	−4.8 (−20.4; 10.8)	0.52	1.4 (−12.0; 14.8)	−0.7 (−17.8; 16.5)	0.85
Food cravings (mm)^a^						
Like to eat something savoury	12.2 (−3.0; 27.4)	9.2 (−10.3; 28.7)	0.80	14.3 (−1.2; 29.8)	1.5 (−18.4; 21.4)	0.31
Like to eat something fatty	19.1 (11.1; 27.1)	20.5 (10.2; 30.7)	0.83	17.1 (5.8; 28.4)	8.8 (−5.6; 23.2)	0.35
Like to eat something sweet	17.9 (6.9; 28.9)	14.9 (0.8; 29.1)	0.74	12.4 (−4.7; 29.5)	16.5 (−5.5; 38.5)	0.77
Like to eat something salty	7.7 (−8.10; 23.4)	12.7 (−7.4; 32.8)	0.69	2.0 (−11.1; 15.1)	10.7 (−6.0; 27.4)	0.40
Health-related Quality of Life						
Physical component score	3.1 (1.2; 4.9)	−0.3 (−2.7; 2.0)	0.03	1.2 (−2.6; 4.9)	0.6 (−4.2; 5.4)	0.84
Mental component score	−1.9 (−4.9; 1.1)	−0.3 (−4.1; 3.5)	0.50	−2.9 (−8.1; 2.3)	−1.2 (−7.8; 5.4)	0.67

^a^An increase in score represents a decrease in perceived desire.

ANCOVA, analysis of covariance; Mitt, modified intention-to-treat.

**Figure 7:**
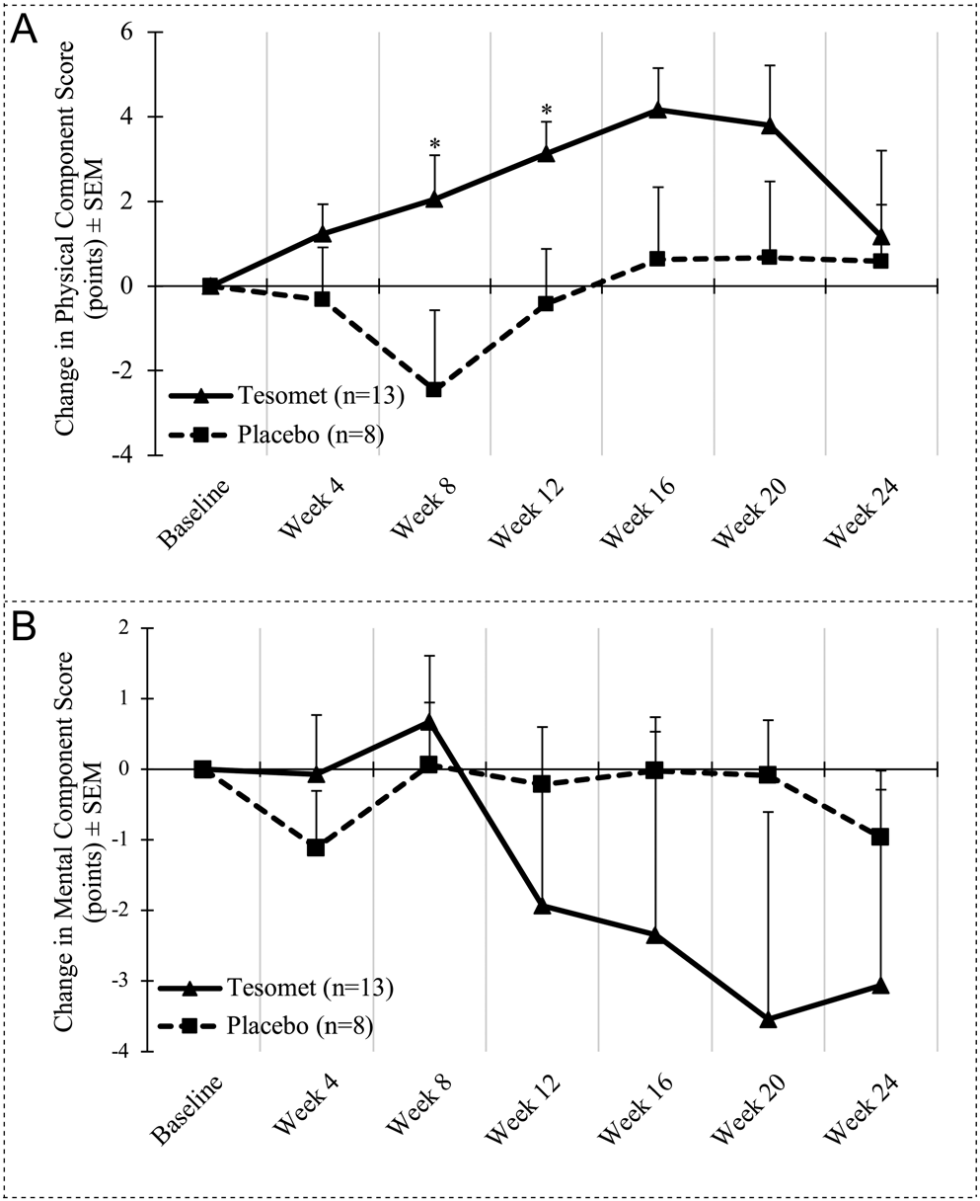
Change in quality of life over time in a randomized controlled trial of Tesomet for weight loss in with hypothalamic obesity. Data are mean change from baseline in physical (A) and mental (B) component scores of SF36 v2 for each treatment group at each scheduled visit (weeks from baseline). Last observation carried forward imputation was used for missing data. Error bars are s.e.m. *P  < 0.05 in a baseline adjusted ANCOVA model with treatment as factor and baseline value as covariate.

Tesomet resulted in numerical improvements in the physical component scores of SF-36 from week 4 to week 20; however, returned to near baseline at week 24, while the placebo group after an initially reduced physical component score at week 8, returned to near baseline at week 16 (Fig. 7A). Both groups had numerical reductions in mental component score to greater extent in Tesomet-treated patients (Fig. 7B).

## Discussion

In this proof-of-concept study, we investigated the effects of Tesomet in adults with hypothalamic obesity. Tesomet was well tolerated and effective in producing sustained and significant mean weight loss of ~7.4 kg (6.6%) over 24 weeks, without increasing blood pressure or heart rate. The placebo group received the same diet and lifestyle counselling, lost ~1.5 kg (1.5%) initially but returned to near baseline at week 24, confirming the limitations of traditional lifestyle weight management (12, 38). Although Tesomet led to significant weight loss and near significant reduction in waist circumference (*P* = 0.052), numerical improvements in fat mass were not significant, probably due to low number of participants and high intra-individual variability, since patients lost more fat (5.3 kg ± 5.3) than lean mass (2.8 kg ± 1.9). Notably, lean mass loss accounted for about 38% of lost weight, which is greater than what is typically observed with diet-induced weight loss (lean mass accounting for 25% of total weight loss) (39). To the extent this greater decrease in lean mass is associated with greater decreases in resting metabolic rate, it is not unreasonable to assume that maintenance of this weight loss may be (more) difficult. However, given the small number of subjects and the large inter-individual variability in this response, we find it premature to draw any solid conclusions about the long-term maintenance of Tesomet-induced weight loss.

The few randomized controlled trials investigating drugs for hypothalamic obesity have reported varying results. An 8-week clinical trial of beloranib, a methionine aminopeptidase 2 inhibitor (beloranib, *n*  = 8) produced mean (95% CI) weight change of −5.0% [−6.6; −3.3] (40). However, beloranib was abandoned due to two fatal adverse events of pulmonary embolism and two adverse events of deep vein thrombosis in a Prader–Willi syndrome phase 3 trial (41). In an open-label study, we previously investigated sibutramine, a monoamine reuptake inhibitor, combined with diet and lifestyle modifications, for 11 months in 14 non-diabetic hypopituitary obese adults and found a mean (s.d.) weight change of –9.9% (4.4) accompanied by reductions in triglyceride concentrations (25). However, sibutramine was withdrawn due to rising concerns of cardiovascular risks (42). Exenatide, a once weekly injected glucagon-like peptide 1 receptor agonist, was investigated in 42 (exenatide, *n*  = 23) adolescents and young adults with hypothalamic obesity. After 36 weeks, the placebo-subtracted change in mean (95% CI) BMI was not significant (−0.8 kg/m^2^ (−1.6; 0.1)) but was significant for fat mass (–3.1 kg (−5.7; −0.4)). Exenatide did not result in improved serum lipids, glucose, or HbA1c over placebo. Six serious adverse events were reported in four patients, including increased seizure activity, altered mental status, migraine, and hypernatremia. Exenatide-related adverse events were otherwise mostly gastrointestinal (43).

Bariatric surgery as treatment for hypothalamic obesity was mostly investigated in case reports or small case–control studies and commonly involved Roux-en-Y gastric bypass, laparoscopic gastric banding, and sleeve gastrectomy. Van Santen *et al.* recently reported that weight loss 5 years after bariatric surgery was less effective in hypothalamic obesity than in general obesity for Roux-en-Y gastric bypass (−22.7% and −32%, respectively, *P*  = 0.003) but comparable for sleeve gastrectomy (−21.7% and −21.8%, respectively, *P*  = 0.96) (14). Following bariatric surgery, generally minor changes in the need for hormonal replacement therapies were reported (14, 44, 45). Importantly, cases of adrenal crises and acute aggravation of diabetes insipidus were reported, which is probably explained by altered post-operative drug absorption (7, 15, 46). Patients also developed the well-known medical and surgical problems that are also common in patients with general obesity (7).

Adverse events were consistent with the pharmacodynamic profile of Tesomet, including frequent occurrence of dry mouth, sleep disturbances, dizziness, and headache. Sleep disturbances reported as insomnia were frequent in patients treated with Tesomet (50%). These were mild (21%) to moderate (29%). Similarly, Astrup *et al.* reported high rates of insomnia (27% of total adverse events) and sleep phase rhythm disturbances (12%) in patients with general obesity treated with 1.0 mg tesofensine (31). Following an audit by the Danish Health and Medicines Authority, concerns were raised regarding the under-reporting of adverse events in particular headache, migraine, stress, and depression in the aforementioned study of tesofensine alone (47). In a response letter, Astrup *et al.* concluded that the listed adverse events were under-reported in all centres which together with inadequate quality of data regarding adverse events with probable or definite relationship to the study drug lead to study discontinuation (48).

Patients with hypothalamic obesity often have sleep disturbances and altered circadian rhythm, possibly due to disturbances in melatonin signalling (7, 49). At randomization, three patients had sleep apnoea (two Tesomet, one Placebo). No new occurrences or worsening of existing sleep apnoea were reported during the trial. Standardized quantification and qualification of sleep disorders were not performed but would be valuable in future trials. Headaches were also more frequently reported in Tesomet compared to placebo (36% vs 0%, respectively). These were mild (29%) to moderate (7%) in intensity, and all resolved spontaneously. None of the adverse events led to changes in Tesomet dose or study discontinuation.

One patient randomized to Tesomet developed severe paranoia and anxiety after ~5–8 weeks of treatment. Investigational treatment was stopped, and the patient was given a sick leave from work to rest after which the patient’s condition improved. The patient had a 5-year history of stress and anxiety, which could have been exacerbated by either component of Tesomet (tesofensine or metoprolol). The patient had no prior experience with either drug. Anxiety is an uncommon (0.01–0.1%) side effect of metoprolol. Tesofensine produces an activation of the monoaminergic systems and could potentially affect mood states and anxiety via receptors in the fronto-limbic neuronal system. Astrup *et al.* measured mood state profiles in healthy obese individuals after daily treatment with 1 mg tesofensine for 24 weeks and did not find any significant changes in total mood disturbance, tension or anxiety, depression or dejection, and fatigue or inertia; however, treatment significantly increased confusion, vigour and activity, anger, and hostility when compared to placebo (31). They also recorded more adverse events related to affective changes (e.g. altered, elevated, and depressed mood) in patients receiving 1 mg tesofensine compared to placebo (24.5% vs 3.8% of total number adverse events, respectively) (31).

Finally, one Tesomet-treated patient had re-growth of craniopharyngioma discovered by a pre-scheduled MRI-scan. The patient underwent intra-cranial surgery and subsequently developed post-procedural complications including transitory aphasia, right hemipalsy, and diabetes insipidus and underwent further neurorehabilitation. Investigational treatment was paused during admission and resumed soon after discharge. Given the re-growth nature of craniopharyngiomas (10-year recurrence rate up to 33%), causality to treatment was unlikely (5).

A contraindication to broad acting monoamine reuptake inhibitors is a potential increase in heart rate and blood pressure, because this exacerbates the already high risk of cardiovascular events and mortality in an at-risk patient group. In this small cohort of adults with hypothalamic obesity, Tesomet did not produce any significant differences in heart rate or blood pressure compared to placebo. This suggests that co-administration of tesofensine and metoprolol can mitigate the previously reported increases in heart rate and blood pressure by tesofensine alone (31). Given that Tesomet shares similarities in its mechanism of action with sibutramine, which was discontinued due to cardiovascular concerns, conclusions regarding safety and tolerability from our study must be carefully considered, and larger investigations are needed to confirm our encouraging preliminary results.

The limitations of this study were mainly related to the small sample size. A placebo-controlled design was chosen to investigate safety and efficacy of Tesomet, but the rarity of hypothalamic obesity combined with strict inclusion criteria to ensure safety and suitability for treatment resulted in a small number of recruited patients. A further bias might be inclusion of mostly White female participants (77%) as hypothalamic obesity does not have a gender predominance (50). Secondly, as the primary endpoint was safety and tolerability, no power calculation for efficacy endpoints were made. Thirdly, the specific type and/or degree of hypothalamic damage and whether this could have influenced weight loss could not be assessed. Lastly, 24 weeks may not be sufficient to demonstrate the full weight reducing potential of Tesomet nor the full spectrum of its potential safety profile. On the other hand, the overall dropout rate was low (18%) and drug compliance WAS high (>90%). This study was thus primarily an exploratory feasibility study as background for a larger controlled trial.

In conclusion, Tesomet at a dose of 0.5 mg tesofensine and 50 mg metoprolol was well-tolerated, did not affect heart rate or blood pressure, and resulted in a sustained progressive weight loss in adults with hypothalamic obesity. These preliminary results are encouraging and support the continued investigation of Tesomet for the treatment of acquired hypothalamic obesity.

## Declaration of interest

U F-R is a member of the Scientific Advisory Board for Saniona A/S. K K and B E are both Senior Medical Advisors to Saniona A/S. B E is a shareholder of Saniona A/S. J D is founder, full-time employee, and shareholder of Saniona A/S. The study was funded by Saniona A/S. An advisory board with representatives from the sponsor and the team of investigators designed the study protocol. All authors approved the manuscript prior to submission. The other authors have nothing to disclose.

## Funding

K H received a scholarship and S B received a PhD scholarship from Saniona A/S for their assistance in conducting the trial.
